# Exceptional point protected robust on‐chip optical logic gates

**DOI:** 10.1002/EXP.20210243

**Published:** 2022-04-04

**Authors:** Song‐Rui Yang, Xu‐Lin Zhang, Hong‐Bo Sun

**Affiliations:** ^1^ State Key Laboratory of Integrated Optoelectronics College of Electronic Science and Engineering Jilin University Changchun China; ^2^ College of Physics Jilin University Changchun China; ^3^ State Key Laboratory of Precision Measurement Technology and Instruments Department of Precision Instrument Tsinghua University Haidian Beijing China

**Keywords:** exceptional point, optical logic gate, robustness

## Abstract

Optical logic gates are crucial components for information processing and communication using photons. Current optical logic gates typically rely on the light interference principle which requires an accurate manipulation of the dynamical phase of light, making the device quite sensitive to system disturbances such as fabrication errors. Here we introduce non‐Hermitian principles into the design of optical logic gates that work in the signal transmission process. We propose an exclusive‐or gate for silicon‐on‐insulator platform by employing the physics in the exceptional point (EP) encirclement process. The EP induced mode switching behavior is applied to manipulate the phase of light which is topologically protected by the energy surface around the EP. As a result, the performance of the device is found to be extremely robust to structural parameter disturbances. The proposed non‐Hermitian principle is expected to find applications for other on‐chip photonic devices toward high robust performance.

## Graphical abstract

Non‐Hermitian physical principles are introduced into the design of on‐chip photonic devices toward high robustness. As a proof‐of‐concept, an exclusive‐or gate on silicon‐on‐insulator platform is designed by employing the exceptional point encirclement physics. The device performance is extremely robust to structural parameter disturbances, owing to the protection of the photon phase by the topological structure around the exceptional point.

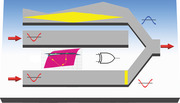

## 1 INTRODUCTION

The use of photons for on‐chip information processing and communication has attracted great attention due to photon's merit of high efficiency, parallel processing capabilities and low latency.^[^ ^1^, ^2^, ^3^, ^4^, ^5^, ^6^, ^7^, ^8^, ^9^, ^10^, ^11^, ^12^, ^13^, ^14^ ^]^ Logical operations are the core issues for information processing, making optical logic gates being crucial components for photonic chips. Currently, there are some ways to realize optical logic gates. The first type is based on linear optical effects, such as Mach‐Zehnder interferometers,^[^ ^15^, ^16^ ^]^ where the dynamical phase of photons in the device should be controlled quite accurately in order to realize the interference condition. As a result, this kind of logic gate has very high requirements on the manufacturing process, since a small disturbance would break the interference effect. The second type of the optical logic gate works based on the nonlinear effect, which however requires special materials and also additional energy input to produce nonlinear effects.^[^ ^17^, ^18^ ^]^ In addition, there are also some other designs of the optical logic gates.^[^ ^19^, ^20^, ^21^, ^22^, ^23^, ^24^ ^]^ However, most of the reported devices are confronted with the problem of low robustness, since the underlying physical mechanism is related to the interference principle which is extremely sensitive to structural disturbances.

Non‐Hermitian physics has received significant attention recently, since the introduction of non‐Hermiticity into Hermitian systems has given rise to many novel phenomena and intriguing applications.^[^ ^25^, ^26^, ^27^ ^]^ Among them, the non‐Hermitian hallmark‐exceptional points (EPs) and their associated topological structures in photonic systems can offer novel ways to manipulate photons such as the chiral transmission of photons via the dynamical encircling of an EP.^[^ ^28^, ^29^, ^30^, ^31^, ^32^, ^33^, ^34^ ^]^ This interesting phenomenon is recently demonstrated successfully in a pure quantum system by Du's group,^[^ ^33^ ^]^ and the dynamics is shown to be topologically protected by the energy surface around the EP. As a result, EPs triggered non‐Hermitian devices are typically robust to system disturbances. Inspired by the rapid progress of non‐Hermitian photonics, introducing this new degree of freedom, that is, non‐Hermiticity, into the design of on‐chip photonic devices such as optical logic gates can broaden the design principle and may lead to novel on‐chip non‐Hermitian devices with high performance.

In this work, we propose the design of optical logic gates based on non‐Hermitian principles and show that the device exhibits a high robustness compared to conventional devices simply based on the light interference effect. We start by studying a three‐state non‐Hermitian system where an EP is dynamically encircled in a subsystem. We find that the chiral dynamics associated with the EP encirclement process can provide a topologically protected phase distribution in the system, which can be used to design an exclusive‐or (XOR) gate. Since the phase distribution required by the XOR gate is protected by the topological structure around the EP, the performance is found to be highly robust to the disturbance of system parameters. This robust non‐Hermitian principle is then applied to the design of an XOR gate for the silicon‐on‐insulator (SOI) platform. The performance of the device is found to be quite insensitive to the change of various device parameters in a wide range. This work provides a robust non‐Hermitian design rule for various photonic devices.

## 2 RESULTS AND DISCUSSION

### 2.1 Theoretical model

**FIGURE 1:**
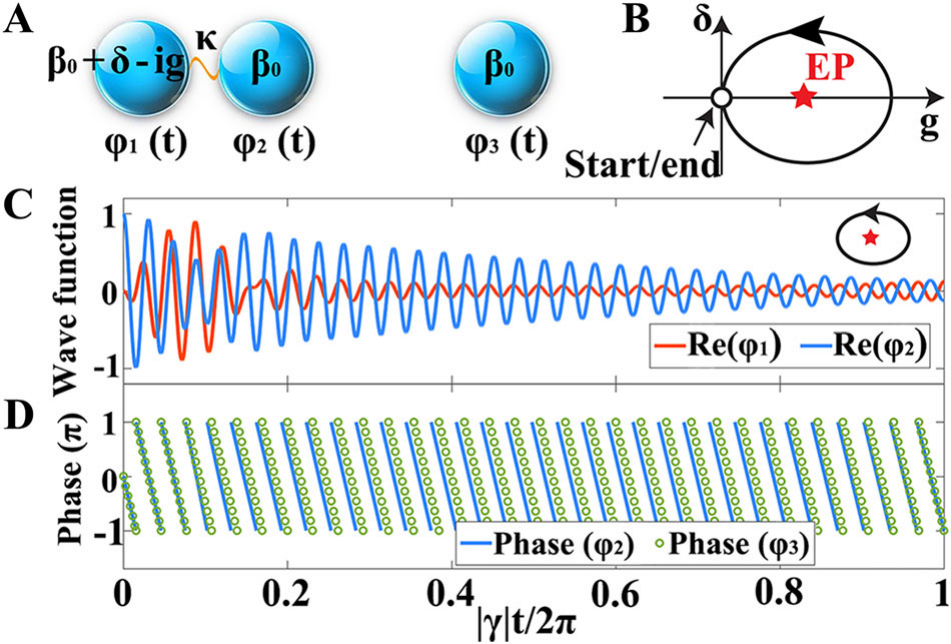
Dynamics in a non‐Hermitian system consisting of three oscillators. (A) Schematic diagram of a three‐oscillator system that evolves in time, where the subsystem consisting of the oscillator‐1 and oscialltor‐2 supports an EP. (B) The δ‐g parameter space in the subsystem where an EP (located at δ=0 and g=2) is dynamically encircled in a counter‐clockwise loop. (C) Calculated real part of the wave functions $\varphi_{1}\;$and $\varphi_{2}$ as a function of time with γ=−0.4. The two wave functions are out of phase at the output side as a result of the EP encirclement. (D) Calculated phase of $\varphi_{2}$ and $\varphi_{3}$ as a function of time with γ=−0.4. They are in phase at the output side. In these calculations, we choose $\beta_{0}=\;13$, κ=1, $g_{0}=\;3$ and $\delta_{0}=\;0.2$

We first propose a theoretical model to demonstrate the design principle behind our non‐Hermitian logic gate. We consider two coupled oscillators in Figure 1A where their wave functions are denoted by $\varphi_{1}\left(t\right)$ and $\varphi_{2}\left(t\right)$, respectively. The on‐site energy of oscillator‐1 is $\beta_{0}+\delta -ig$, where δ is a detuning parameter and g indicates that the wave function of oscillator‐1 is decreasing with time. The on‐site energy of oscillator‐2 is $\beta_{0}$ and the coupling between the two oscillators is denoted by κ. The dynamics of this non‐Hermitian system is governed by a Schrodinger‐like equation $\hat{H}\;\left(\begin{array}{c} \varphi_{1}\left(t\right)\\ \varphi_{2}\left(t\right) \end{array}\right)=\;i\frac{\partial }{\partial t}\left(\begin{array}{c} \varphi_{1}\left(t\right)\\ \varphi_{2}\left(t\right) \end{array}\right)$, where the model Hamiltonian reads

(1) $$\begin{array}{c} \hat{H}=\left(\begin{array}{cc} \beta_{0}+\delta -ig & \;\kappa \\ \kappa & \;\beta_{0} \end{array}\right). \end{array}$$

Without loss of generality, we set κ=1 and an EP can be found in the system at δ=0 and g=2, corresponding to the simultaneous coalescence of eigenvalues and eigenfunctions. We will show that by a dynamical encirclement of this EP, we can manipulate the phase in the two oscillators, which is in fact the key to the design of the logic gate.

Figure 1B shows the g−δ parameter space where we have designed a loop that encloses the EP with the formula $\delta \;=\delta_{0}\;\sin\left(\gamma t\right)$ and $g\;=g_{0}\;\left(1-\cos(\gamma t)\right)$. Here γ is a parameter that measures the adiabaticity of the looping process. By substituting them into the Hamiltonian equation, we can obtain the equation governing the dynamics of $\varphi_{1}$ and $\varphi_{2}$, for example,

(2) $$\begin{array}{c} \frac{\partial^{2}\varphi_{2}\left(t\right)}{\partial t^{2}}+\left[2i\beta_{0}+i\delta_{0}\sin\left(\gamma t\right)+g_{0}\left(1-\cos\left(\gamma t\right)\right)\right]\frac{\partial \varphi_{2}\left(t\right)}{\partial t}\\ \;+\;\left[i\beta_{0}g_{0}\left(1-\cos\left(\gamma t\right)\right)-\beta_{0}\delta_{0}\sin\left(\gamma t\right)+1-\beta_{0}^{2}\right]\varphi_{2}\left(t\right)=0. \end{array}$$

During the evolution of the system over time, that is, t=0∼2π/|γ|, the operator$\;\hat{H}\;$will finally return to the original one as the system goes around the EP, but the wave functions may change due to the topological structure around the EP. To show this point, we plot the calculated wave functions in Figure 1C with γ=−0.4, corresponding to a counter‐clockwise loop to encircle the EP (see Figure 1B for the loop). The initial condition is chosen as $\varphi_{1}\;\left(t_{0}\right)=\;0$ and $\varphi_{2}\;\left(t_{0}\right)=\;1$. We find that at the final time step ( $t_{\mathrm{end}}=\;2\pi /\left|\gamma \right|$), there is a phase difference of π between $\varphi_{1}\left(t_{\mathrm{end}}\right)$ and $\varphi_{2}\left(t_{\mathrm{end}}\right)$. This is a result of the dynamical encirclement of the EP,^[^ ^28^ ^]^ and such result is independent of the system adiabaticity (i.e., γ), input state vector (i.e., $\varphi_{1}\left(t_{0}\right)$ and $\varphi_{2}\left(t_{0}\right)$), and the looping trajectory (i.e., detailed formula of g and δ) as long as the EP is encircled. We will show later that this phase difference of π is key to our device, since manipulating the wave phase is crucial for the design of logic gates.

Having studied the subsystem consisting of the oscillator‐1 and oscillator‐2, we study another subsystem with oscillator‐3 only (see Figure 1A). The two subsystems do not couple with each other, but they form a whole system that can realize the logic functionality. The wave function of oscillator‐3 is represented by $\varphi_{3}$ which satisfies $\beta_{0}\varphi_{3}\left(t\right)\;=\;i\frac{\partial }{\partial t}\varphi_{3}\left(t\right)$ where the on‐site energy of oscillator‐3 is $\beta_{0}$. We solve the evolution of $\varphi_{3}\left(t\right)$ in the same time frame (i.e., t=0∼2π/|γ|) with the initial condition $\varphi_{3}\;\left(t_{0}\right)=\;1$ and show the phase of $\varphi_{3}\left(t\right)$ in Figure 1D. The phase of $\varphi_{2}\left(t\right)$ is also given for comparison. We find that although the phase of $\varphi_{2}\left(t\right)$ and $\varphi_{3}\left(t\right)$ differ during the evolution process, they are the same at the final time step. This is not by coincidence but resulted from the fact that the oscillator‐2 and oscillator‐3 share the same on‐site energy which mainly determines the phase accumulation over time. Since there is a phase difference of π between $\varphi_{1}\left(t_{\mathrm{end}}\right)$ and $\varphi_{2}\left(t_{\mathrm{end}}\right)$, we can reach a key conclusion in our system that $\varphi_{1}\left(t_{\mathrm{end}}\right)$ and $\varphi_{3}\left(t_{\mathrm{end}}\right)$ will have a π phase difference. This can be applied to design an XOR gate in which the interference between $\varphi_{1}\left(t_{\mathrm{end}}\right)$ and $\varphi_{3}\left(t_{\mathrm{end}}\right)$ is used as the output, while $\varphi_{2}\left(t_{0}\right)$ and $\varphi_{3}\left(t_{0}\right)$ are the two inputs. Based on this configuration, the truth table of the system is given in Table 1. We should emphasize that the direction to encircle the EP is crucial for realizing the desired functionality, since different encircling directions lead to different final states. This is in fact the reason that we choose a counter‐clockwise loop in the above model, whereas the clockwise‐loop physics cannot be applied for the design (see Supporting Information and Figures S1 and S4 for detailed discussions).

**TABLE 1:** Truth table of the designed XOR gate, where “0” means a signal with a power below the threshold, while “1” means that above the threshold

Input A: $\varphi_{2}\left(t_{0}\right)$ in model or waveguide 2 in device	Input B: $\varphi_{3}\left(t_{0}\right)$ in model or waveguide 3 in device	Output Y: Interference between $\varphi_{1}\left(t_{\mathrm{end}}\right)$ and $\varphi_{3}\left(t_{\mathrm{end}}\right)$ in model or interference between waveguides 1 and 3 in device
0	0	0
0	1	1
1	0	1
1	1	0

**FIGURE 2:**
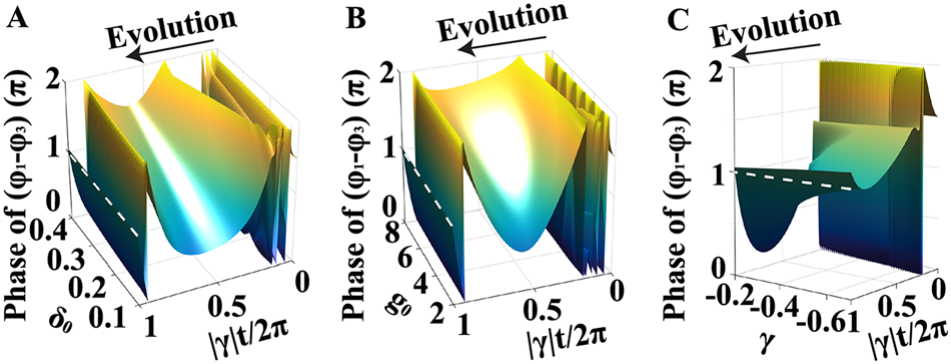
Robustness of the XOR gate based on the three oscillators. (A–C) The calculated phase difference between$\;\varphi_{1}$ and$\;\varphi_{3}\;$during the evolution with varying (A) $\delta_{0}$, (B) $g_{0}$, and (C) γ. Other parameters are fixed at (A) $g_{0}=\;3$ andγ=−0.1, (B) $\delta_{0}=\;0.2$ and γ=−0.1, and (C)$\;\delta_{0}=\;0.2$ and $g_{0}=\;3$. The white dashed line marks the region with $\varphi_{1}-\;\varphi_{3}=\;\pi$

Although the designed XOR gate works based on the interference principle, the key phase difference (i.e., $\varphi_{1}\left(t_{\mathrm{end}}\right)-\varphi_{3}\left(t_{\mathrm{end}}\right)$) is topologically protected since it is realized by the EP encirclement process. Therefore, the functionality is robust to the variation of system parameters. To verify this, we calculate $\varphi_{1}\left(t\right)-\varphi_{3}\left(t\right)$ by changing the values of$\;\delta_{0}$,$\;g_{0}\;$andγ, respectively, in Figure 2A–C. We find that although the phase difference is irregular in the evolving process, it is always ∼π at the final time step in Figure 2A and B, indicating that the loop trajectory would not affect the phenomenon. By increasing the absolute value of γ, the phase difference goes away from π since the adiabaticity gets worse. Therefore, the phase effect is extremely robust as long as the system is adiabatic enough, which means that the proposed XOR gate will also be topologically robust.

### 2.2 Device design

**FIGURE 3:**
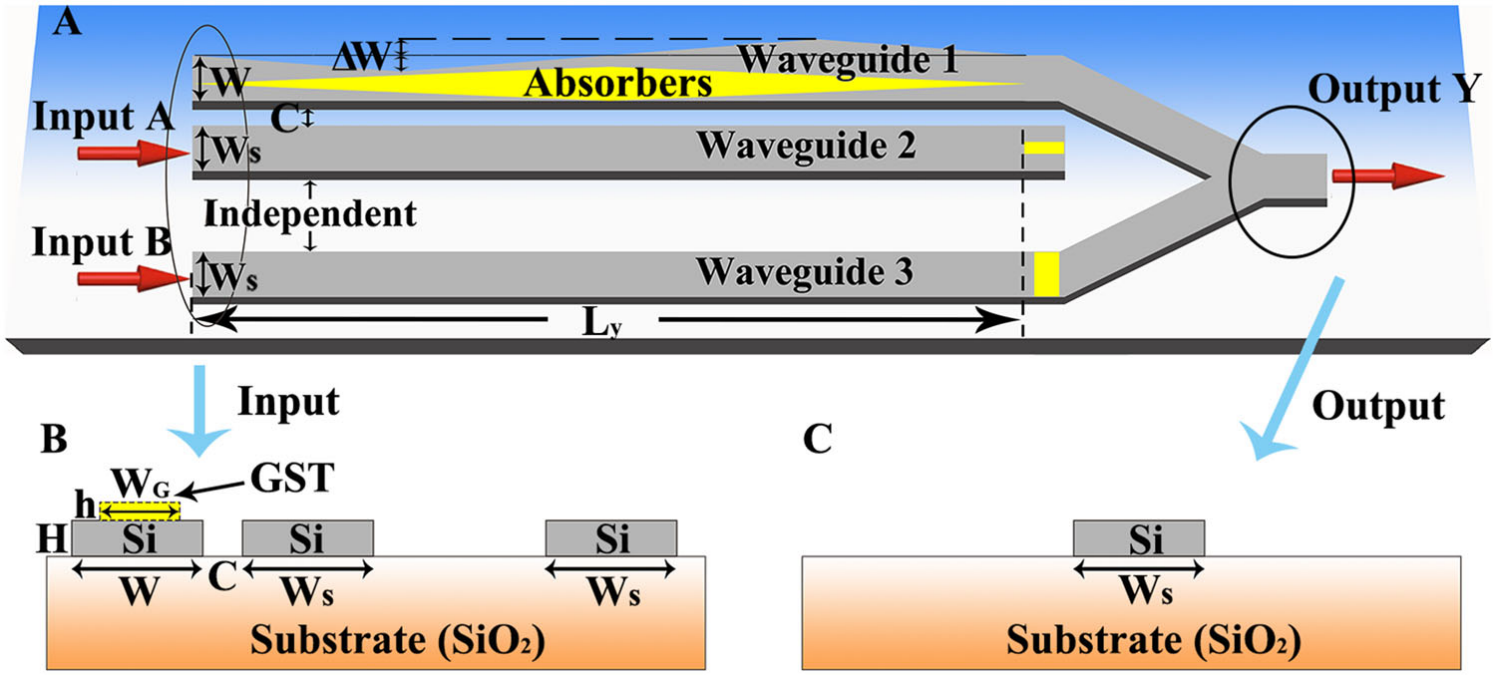
Design of an XOR gate on the SOI platform. (A) Schematic diagram of an optical XOR gate based on the SOI platform, where the silicon waveguide‐1 with a varying width and a GST absorber attached can couple with the uniform waveguide‐2, while the uniform waveguide‐3 is independent from them. The two input terminals of waveguide‐2 and waveguide‐3 are termed as port A and port B, respectively, which are used as the input of the logic gate. The waveguide‐1 and waveguide‐3 are merged together at the output port Y. In the design, we choose *L*_y_ = 50 μm. The cross section of (B) input and (C) output side of the waveguide system

We apply the EP‐encirclement principle to design an XOR gate for the SOI platform. We use silicon waveguides to mimic the oscillator in the model since electromagnetic waves propagating in waveguides follow the Schrodinger‐like equation so that the required Hamiltonian can be constructed. Figure 3A shows the schematic of the three‐waveguides system, where the waveguide‐*k* is designed to mimic the functionality of oscillator‐k in the theoretical model (*k* = 1, 2, 3). In this way, the waveguide‐1 and waveguide‐2 can couple together with a gap distance C = 155 nm, while the waveguide‐3 is far away from them to ensure its independence. The cross‐section size of waveguide‐2 and waveguide‐3 is *W*_S_ = 655 nm and *H* = 100 nm (also see Figure 3B), while the width of waveguide‐1 is denoted by W which varies continuously along the waveguiding direction (i.e., y‐axis) in order to reproduce the effect of δ in the model Hamiltonian. To introduce the effect of g, waveguide‐1 is covered by an absorbing layer Ge_2_Sb_2_Te_5_ (short for GST, see the yellow area). The GST is designed to work at its crystalline state with a refractive index of ∼7+2i at the working wavelength of 1550 nm.^[^ ^35^ ^]^ The height of GST is fixed at 2 nm and its width is a function of y in order to meet the requirement of the model Hamiltonian. The introduction of GST at the end of waveguide‐2 is to transfer residual energy from waveguide‐2 to waveguide‐1. The GST at the end of the waveguide‐3 is to match its energy with that in waveguide‐1 in order to ensure an interference in the output waveguide.

**FIGURE 4:**
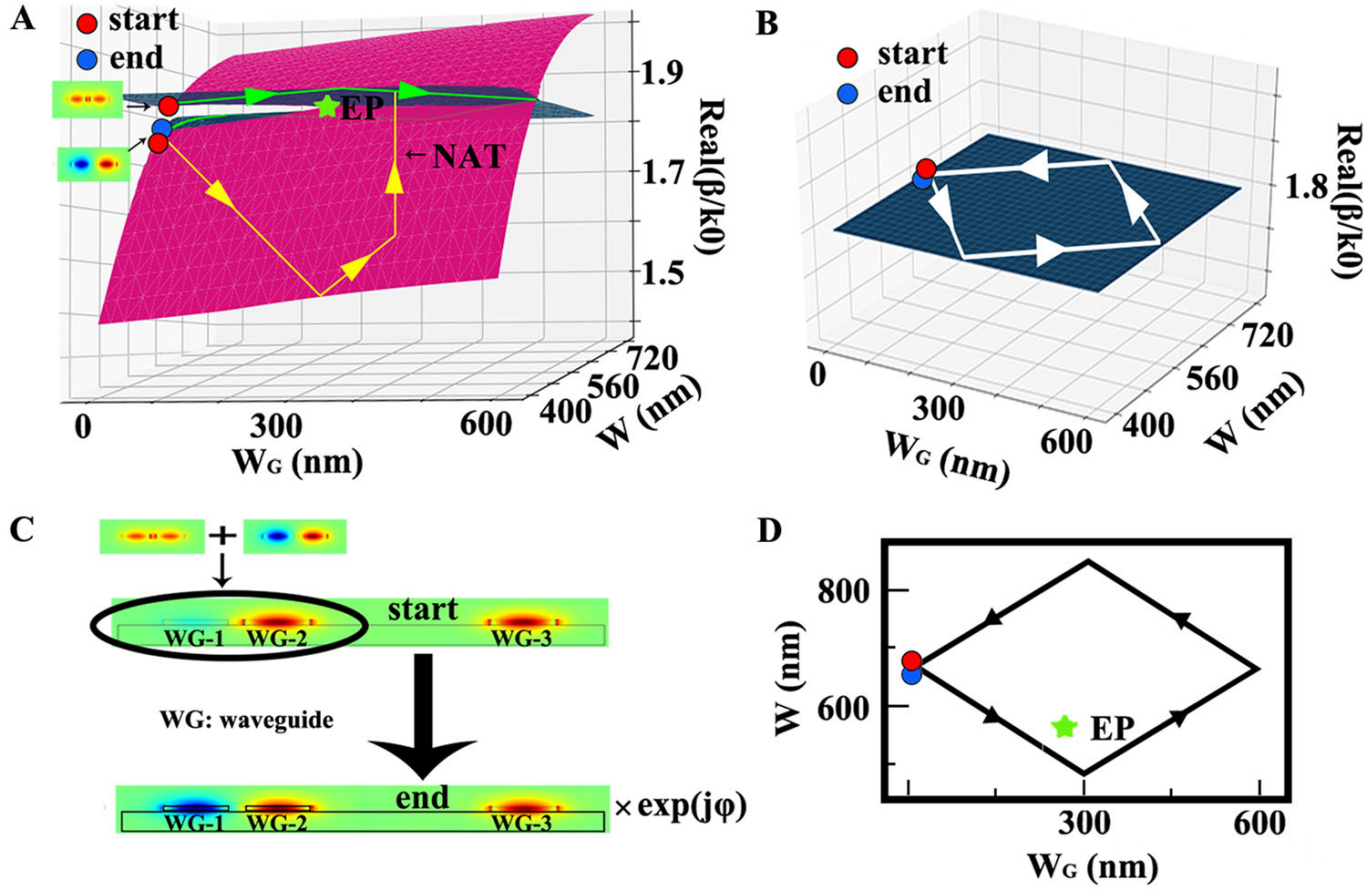
Non‐Hermitian physics in the XOR gate. (A) Calculated real part of the propagation constants of the subsystem consisting of waveguide‐1 and waveguide‐2 as a function of *W* and *W*_G_. The green trajectory shows the EP encircling process starting from the symmetric mode, while the yellow trajectory marks that starting from the antisymmetric mode which possesses a NAT. As a result, the final state is always the antisymmetric mode with a lower β marked by the blue circle. (B) Calculated real part of the propagation constants of the subsystem consisting of waveguide‐3 only. The white trajectory represents the evolution process where only a dynamical phase is accumulated. (C) Evolution of the waveguide modes in the two subsystems. At the starting point, two in‐phase signals are injected through waveguide‐2 and waveguide‐3, respectively. At the end region, the waves in waveguide‐1 and waveguide‐3 are out of phase as a result of the EP encirclement. (D) Parameter space and the loop enclosing the EP in the subsystem consisting of waveguide‐1 and waveguide‐2

As inspired by the model Hamiltonian, we use waveguide‐2 and waveguide‐3 as the two input terminals of the XOR gate, which are termed as input port A and input port B, respectively. At the output side, the waveguide‐1 and waveguide‐3 are merged to form a single waveguide which is used as the output port Y. We first consider the subsystem consisting of waveguide‐1 and waveguide‐2. Figure 4A plots the calculated real part of the propagation constants β as a function of *W* and *W*_G_ in this subsystem, where an EP is found to be located at *W* ≈ 560 nm and *W*_G_ ≈ 290 nm. The simulation was performed using COMSOL.^[^ ^36^ ^]^ The blue (red) energy sheet represents the eigenstate with a lower (higher) loss. Electromagnetic waves transmission in the subsystem leads to a process encircling the EP in a counter‐clockwise loop in the *W*‐*W*_G_ parameter space (also see Figure 4D). At the starting point of the loop (i.e., *W* = 655 nm and *W*_G_ = 0), the two eigenstates supported are symmetric (with a larger β) and antisymmetric states (with a smaller β), since the waves in the two waveguides are in phase and out of phase, respectively. In this way, injecting a wave purely through waveguide‐2 corresponds to a simultaneous excitation of these two eigenstates. However, the final state after encircling the EP is independent of the input state, although the evolution with the symmetric input is adiabatic (see the yellow trajectory in Figure 4A) while that with the antisymmetric input shows a nonadiabatic transition (NAT, see the green trajectory and the NAT in Figure 4A). In the proposed subsystem, the output is the antisymmetric mode, and the phase difference (∼π) between waveguide‐1 and waveguide‐2 is topologically protected by the EP.

The process when the waveguide‐3 mode is excited is shown in Figure 4B. Since the corresponding propagation constant is not affected by *W* an*d W*_G_, only an accumulation of the dynamical phase occurs in the evolution process. Since the waveguide‐2 and waveguide‐3 are identical, the dynamical phases accumulated inside them are almost equal so that at the output side, the waves in the waveguide‐1 and waveguide‐3 are out of phase (see Figure 4C), giving rise to a destructive interference and therefore the truth table of the XOR gate in Table 1.

### 2.3 Robustness of the device performance

**FIGURE 5:**
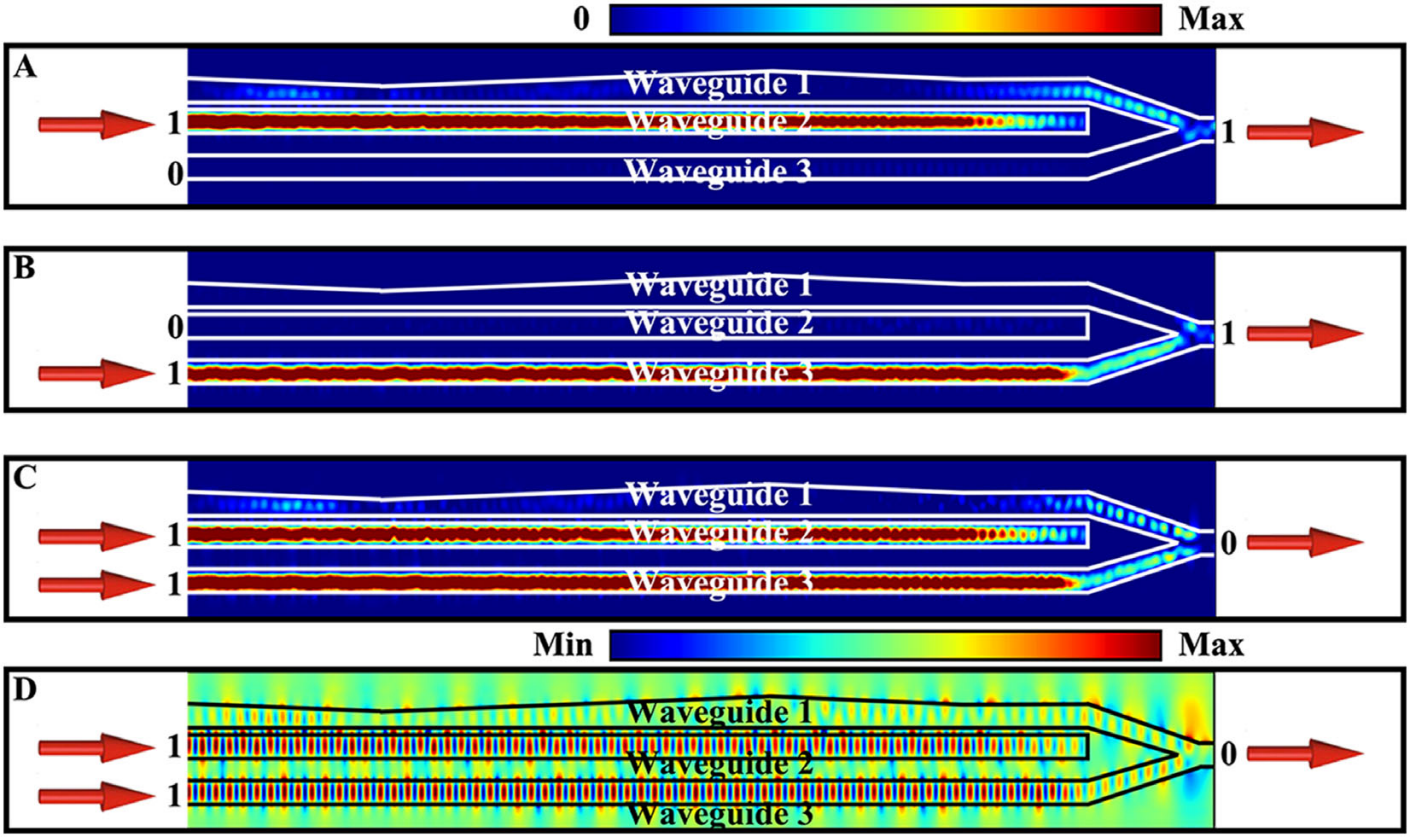
Performance of the XOR gate. The power flow distributions in the device when (A) port A, (B) port B, and (C) dual ports are excited, respectively. These results clearly demonstrate the truth table of the XOR gate. (D) The electric field distributions in the device when dual ports are excited, where the destructive interference at the output port is protected by the EP encirclement process. The wavelength is chosen at 1550 nm for all the simulations

We calculated the wave scatterings in the device to verify the above design. All the four cases in the truth table are analyzed. The first case is obviously satisfied since the output is zero when there is no incidence. When there is only one signal injected through either the waveguide‐2 or the waveguide‐3, the output signal is simply “1”, as shown by the power flow distributions in Figure 5A and B, respectively. The key to the XOR gate is the case with dual injections. The corresponding power flow and electric field distributions are given in Figure 5C and D, respectively. We find that in accordance with the design principle, the electric fields at the output side of waveguide‐1 and waveguide‐3 are out of phase as a result of the EP encirclement process. This phase difference of π induces a destructive interference in the output waveguide, and therefore the output signal is “0”.

**FIGURE 6:**
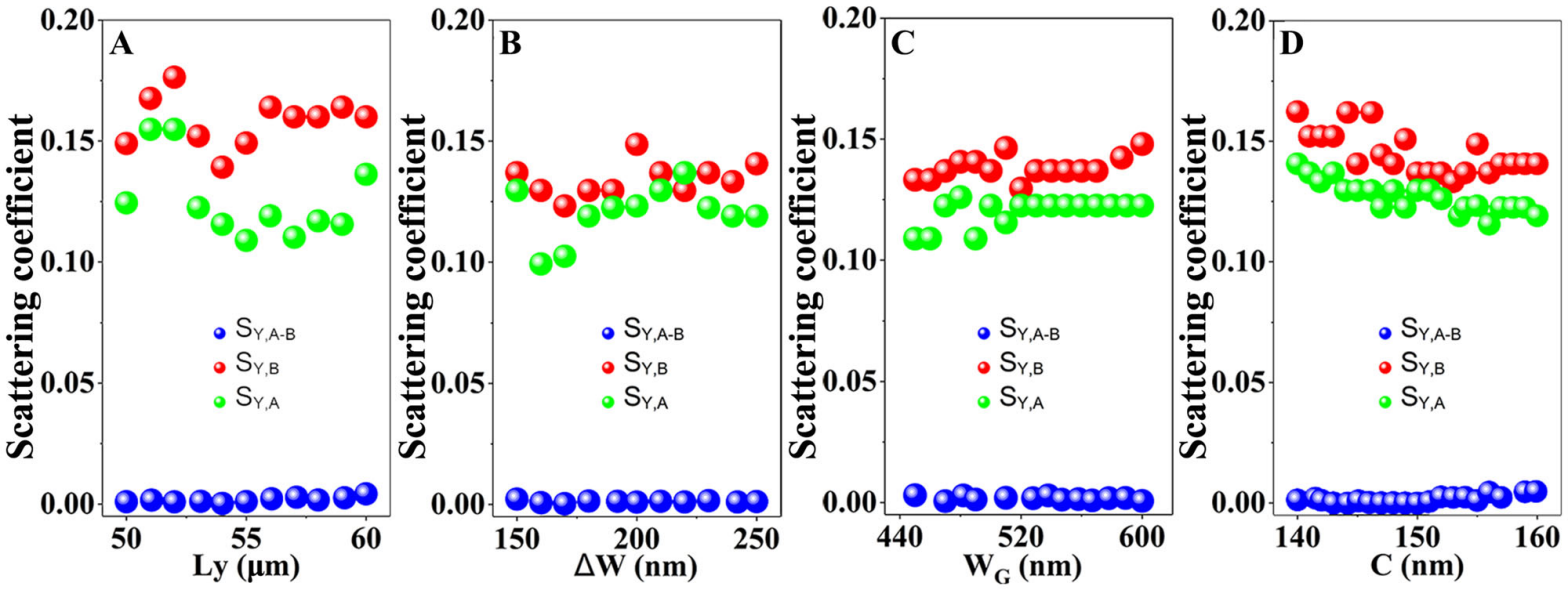
Robustness of the XOR gate. (A–D) Calculated scattering coefficients in the logic gate when (A) the device length *L*_y_, (B) the maximum varying width of the waveguide‐1 ΔW, (C) the maximum width of the GST *W*_G_, and (D) the gap distance C are changing in a wide range. The functionality of the XOR gate is extremely robust to the disturbance of these parameters

We have realized the functionality of an on‐chip optical logic gate. Since the underlying physical mechanism is based on the EP encirclement effect which can provide a topologically protected phase relation, the XOR gate should be quite robust to the change of system parameters. This is in stark contrast to previous optical logic gates based on dynamical phase effect which is however quite sensitive to parameter disturbances. To prove this point, we change the parameters of the system to test the robustness. We choose four key parameters of the system including the gap distance C, the maximum detuning width of waveguide‐1 ΔW, the device length *L*_y_, and the maximum width of GST *W*_G_. Figure 6A–D shows the calculated scattering coefficients with different inputs by varying these four system parameters in a wide range. The scattering coefficient S_Y,A_ (S_Y,B_) is defined as the transmission from port A (port B) to port Y (i.e., the ratio between the output power and the input power), while S_Y,A‐B_ denotes the transmission with dual injections.

We find that such larger disturbances to the system actually do not affect the functionality of the logic gate, since the required output signal of “0” and “1” can be clearly distinguished by choosing a proper threshold. At this point, we can conclude with certainty that the EP‐based optical XOR gate exhibits a robust performance owing to the topological structure‐protected phase. This is in stark contrast to logic gates simply based on the interference effect where the performance is quite sensitive to parameter disturbances (see Figure S5 for an example). This non‐Hermitian principle shows great potential for designing other types of photonic devices with high robustness. For example, if one of the two input terminals always has a signal input in the current design, our XOR gate can simply be turned into a NOT gate. The proposed non‐Hermitian scheme is also highly scalable and can be extended to design devices working at other frequencies such as Terahertz frequencies (e.g., by introducing Graphene to induce the EP at this frequency) or Microwave frequencies (e.g., by adding magnetic absorbers to make the system to be non‐Hermitian).

## 3 CONCLUSION

To conclude, we have studied a non‐Hermitian system consisting of three harmonic oscillators which evolve in time to mimic the dynamical encircling of an EP in a designed parameter space. A topologically protected phase relationship was found which can be used to design an XOR gate. Based on this non‐Hermitian principle, a SOI platform based optical XOR gate was proposed. Compared with previous optical logic gates that are quite sensitive to the variation of structural parameters, the non‐Hermitian logic gates employing the physics of the EP exhibit strong robustness to system disturbances, owing to the undisturbed phase protected by the topological structure of the EP. We emphasize that the proposed device in this work is used in the process of signal transmission or processing where only the optical signals are manipulated. To achieve a logic functionality on current photonic chips, additional photoelectric conversion components are still needed. The non‐Hermitian principles are expected to find more applications for on‐chip photonic devices.

## CONFLICT OF INTEREST

The authors declare no competing interests.

## Supporting information

Supporting Information
