# Supplementary material for: Exceptional point protected robust on‐chip optical logic gates
Source: Exploration (Beijing). 2022 Apr 4;2(3):20210243. doi: 10.1002/EXP.20210243 (PMC10191016; doi:10.1002/EXP.20210243)
Supplement: Supplementary file 1 — Supporting Information [file EXP2-2-20210243-s001.docx]

Supporting Information for

**Exceptional point protected robust on-chip optical logic gates**

*Song-Rui Yang,^1,2^ Xu-Lin Zhang,^1,*^ and Hong-Bo Sun^1,3,*^*

1. The choice of the direction to encircle the EP

Here we give the reason why we choose a loop that encircles the EP in a counter-clockwise direction in Figure 1 of the main text. In fact, there are two ways to encircle the EP: clockwise and counter-clockwise directions. In the main text, we give the results of encircling the EP in counter-clockwise loops in Figure 1 of the main text. Here for completeness, we give the results of clockwise loops in Figure S1. We can see that the final state for clockwise loops is always a symmetric mode, i.e., the wave functions in oscillator-1 and oscillator-2 are in phase (see Figure S1A). As a result, the wave functions in oscillator-1 and oscillator-3 at the final step are in phase. In this way, the destructive interference condition can no longer be satisfied so that this configuration cannot be used to construct an XOR gate. This is the reason that we choose to encircle the EP in counter-clockwise directions in the main text to construct the XOR gate. This principle also applies to the design of the XOR gate based on the SOI platform as shown in Figure 3 of the main text.

The above analysis indicates that the counter-clockwise loop can give a robust phase difference at the final time step between the three oscillators, which is the key to the design of the logic gate. In the main text, we have shown the phase of $\varphi\left( t_{end} \right)$between oscillator-1 and oscillator-3. Here for completeness, we show the phase of $\varphi\left( t_{end} \right)$between oscillator-2 and oscillator-3. During the evolution of the system, the phase in oscillator-2 and oscillator-3 are different, but at the final time step,the phase in oscillator-2 and oscillator-3 are always the same. The corresponding results are shown in Figure S2, which indicates that we have successfully bounded the phases of the wave functions of oscillators-1 and oscillators-2.

1. **Additional results in the numerical design**

Under non-Hermitian conditions, the eigenvalues of the system are complex values. In the main text, we only show the real part of the eigenvalue of the system in Figure 4A of the main text. Here we show the imaginary part of the eigenvalues of the system in the W-W_G_ parameter space in Figure S3. We can also find the same EP in this parameter space.

We also consider the case of encircling the EP in a clockwise direction in the SOI structure. The theoretical model indicates that such a system cannot be used for the design of an XOR gate. The key to the XOR gate is actually the process with dual injections. Therefore, we have performed another simulation for this case in Figure S4 by changing the width variation of waveguide-1. In this simulation, the width of waveguide-1 is increased at first, which is just opposite to the structure in Figure 3 of the main text. In this way, the EP is encircled in a clockwise loop. The results in Figure S4 indicate that the final state for the clockwise loop in the subsystem consisting of waveguide-1 and waveguide-2 is a symmetric mode, which induces a constructive interference at the output port Y. That means when the signal of port A and port B are both “1”, the port Y is also “1”. It can be found in the electric field phase distribution (see Figure S4B) that the phases of the signals in the two waveguides at the output port are the same at this time. Clearly, this is not the functionality of an XOR gate. Therefore, the direction to encircle the EP is very important for the design of the logic gate.

**Supporting Figures**


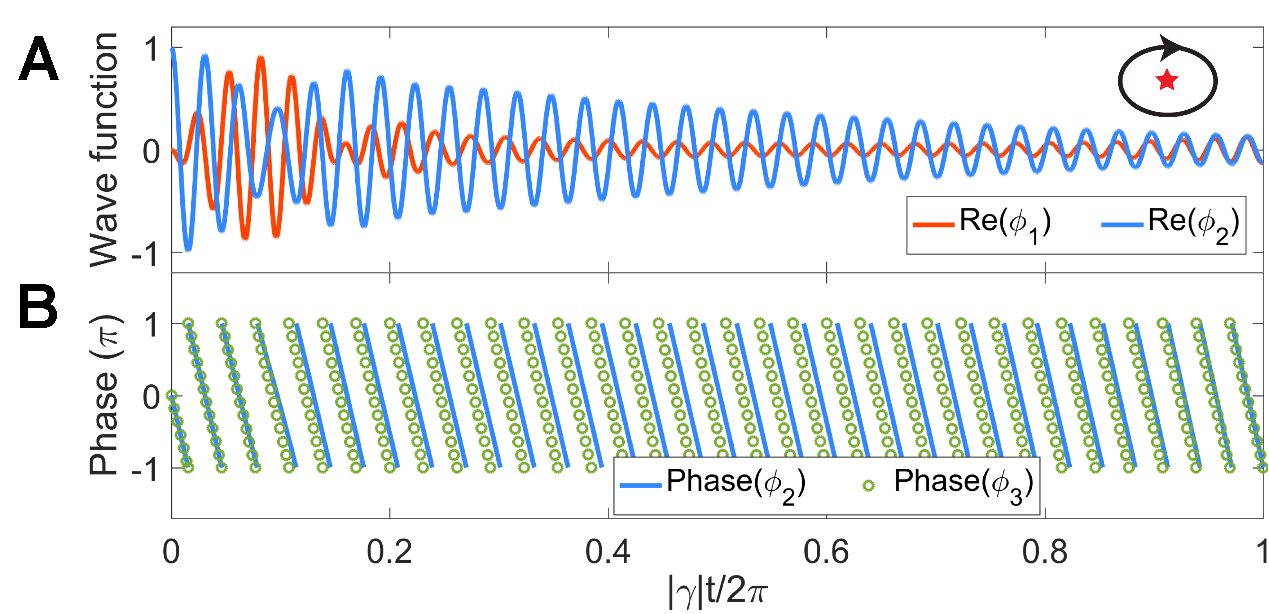


**FIGURE S1** (A) Calculated real part of the wave functions $\varphi_{1}$ and $\varphi_{2}$ as a function of time with $\gamma=-0.4$, when the EP is dynamically encircled in a clockwise direction. The two wave functions are out of phase at the output side as a result of the EP encirclement. (B) Calculated phase of $\varphi_{2}$ and $\varphi_{3}$ as a function of time with $\gamma=-0.4$. They are in phase at the output side. In these calculations, we choose $\beta_{0}=13$ and $\kappa=1$ without loss of generality.


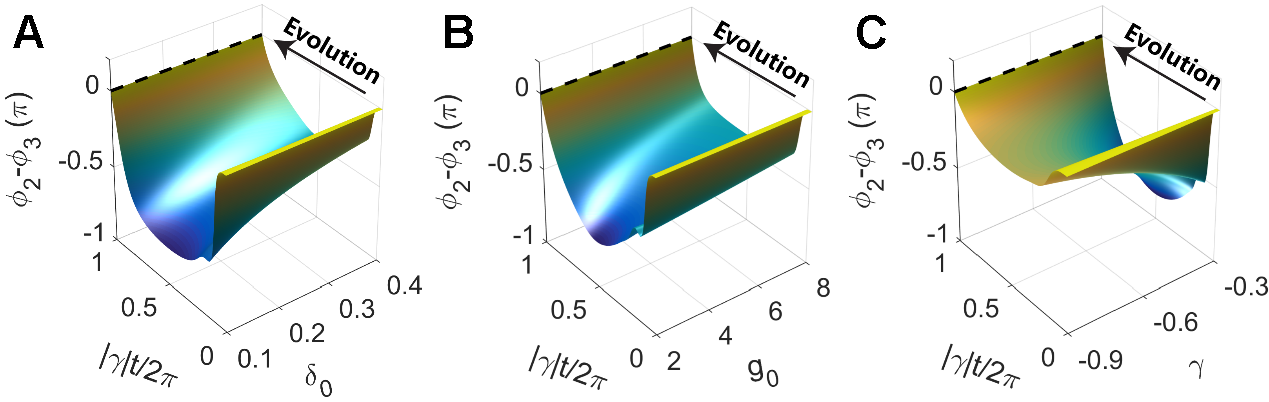


**FIGURE S2** The calculated phase difference between$\varphi_{2}$and$\varphi_{3}$during the evolution with varying$\delta_{0}$ (A), $g_{0}$(B) and$\gamma$ (C). Other parameters are fixed at$g_{0}=3$ and$\gamma=-0.1$ (A), $\delta_{0}=0.2$ and $\gamma=-0.1$ (B), and$\delta_{0}=0.2$ and $g_{0}=3$ (C). The black dashed line marks the region with $\varphi_{2}-\varphi_{3}=0$.


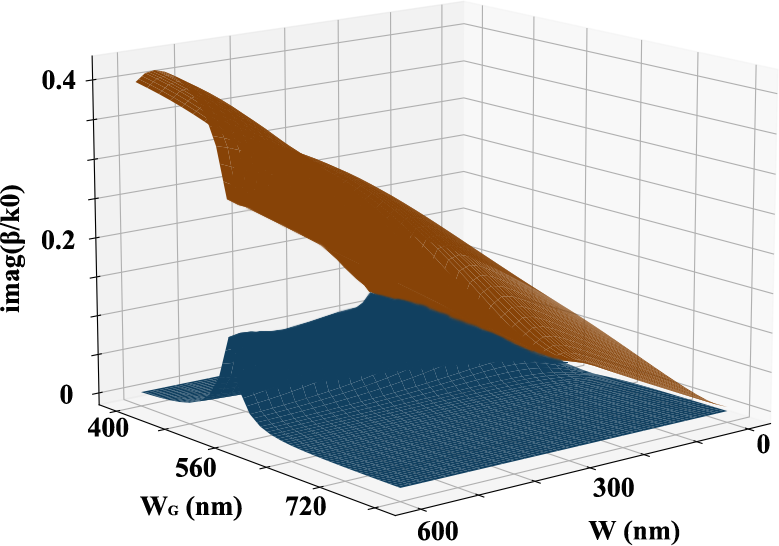


**FIGURE S3** Calculated imaginary part of the propagation constants of the subsystem consisting of waveguide-1 and waveguide-2 as a function of W and W_G_. The blue sheet and the red sheet represent the eigenstate with a lower and higher loss, respectively. As a result of the dynamical encircling of the EP in a counter-clockwise direction, the final state is always the antisymmetric mode with a smaller $\beta$.


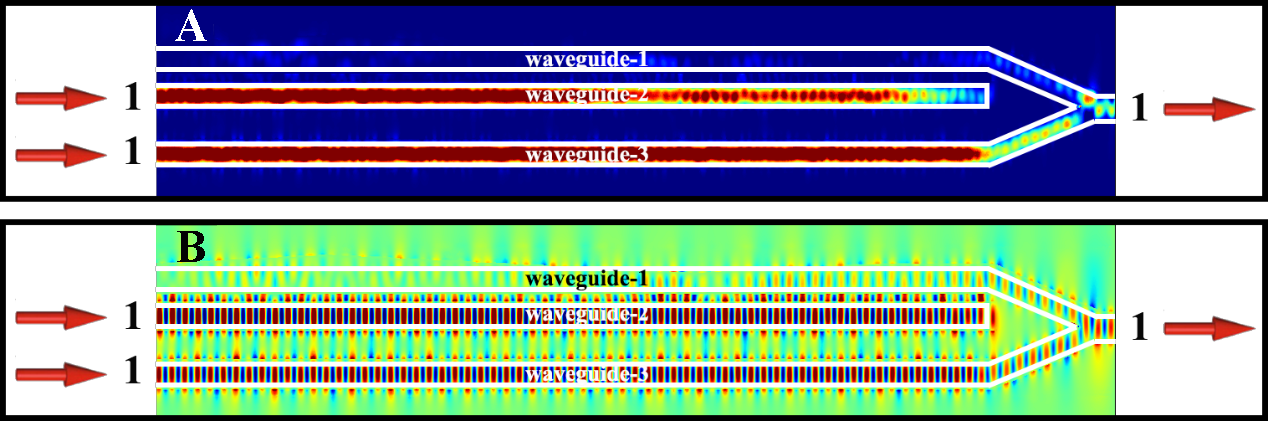


**FIGURE S4** (A and B) Power flow distributions (A) and electric field distributions (B) in the structure by injecting two signals with the same phase via waveguide-2 and waveguide-3, where the EP is encircled in a clockwise direction, which is just opposite to the case in the main text. The output also shows strong signals so that this configuration cannot be used to the design of an XOR gate. This indicates that the encircling direction is crucial to realize the logic functionality.
